# Primary Gastric Burkitt’s lymphoma:

**DOI:** 10.12669/pjms.335.13002

**Published:** 2017

**Authors:** Adnan khan, Sarbiland khan, Umair Arshad

**Affiliations:** 1 Dr. Adnan Khan, House Officer, Rehman Medical Institute, Peshawar, Pakistan; 2 Sarbiland Khan, House Officer, Rehman Medical Institute, Peshawar, Pakistan; 3 Umair Arshad, House Officer, Rehman Medical Institute, Peshawar, Pakistan

**Keywords:** Burkitt’s lymphoma, Stomach, NHL

## Abstract

The primary gastrointestinal non-Hodgkin’s lymphoma is a rare entity. Burkitt’s lymphoma (BL) is an aggressive form of B-cell lymphoma which is endemic in Africa, while in rest of the world non-endemic cases has been reported. Primary gastric BL is extremely rare and only around 53 cases have been reported till now. Here we present the case of a middle-aged male, immunocompetent who presented with anorexia weight loss and diarrhea. His upper gastrointestinal endoscopy and biopsy revealed a large primary gastric Burkitt lymphoma. After chemotherapy, he remains in remission.

## INTRODUCTION

Burkitt’s lymphoma, a disease frequently found in children of Tropical Africa, was first described by an Irish surgeon “Denis Burkitt” in Kampala, Central Africa in the mid-1900s. It was initially believed to be a sarcoma, frequent in children of Tropical Africa.^1^,^2^ In 1961, British pathologist and academic by the name of Michael Anthony Epstein discovered a particular virus in tissue samples he took from this lymphoma. He named this virus as Epstein-Barr virus (EBV). This was the first time a viral pathogen was found to be involved in a human tumor. Burkitt’s lymphoma remains to be the most frequent childhood malignancy in Africa to date.^3^

According to the classification given by the World Health Organization, there are three types of Burkitt’s lymphoma based on clinical grounds; Endemic, sporadic and immunodeficiency associated. Nearly all cases are caused by EBV. Endemic Burkitt’s affects children between 4-7 years of age, affect males more with a 2:1 male to female ratio. Sites affected include; the bones of the jaw and face, the gastrointestinal tract, the kidneys, the ovaries, breasts, and other extra nodal sites.^4^ In Africa, endemic Burkitt’s has the highest incidence.^5^

Sporadic Burkitt’s lymphoma occurs globally. It accounts for 1-2% of all adult lymphomas and 40% of all childhood lymphomas in the U.S and western Europe.^5^ The sites most commonly affected are; the abdomen, the ileocecal region, the ovaries, the kidneys, omentum, Waldeyer’s ring and the central nervous system.^5^ The central nervous system (CNS) involvement at presentation has been reported in 13-17% of the sporadic cases Immunodeficiency virus-associated BL occurs mainly in patients infected with HIV. BL accounts for 30-40% of non-Hodgkin’s lymphoma in HIV-positive patients.^5^

## CASE PRESENTATION

A 45 years old male presented with a one-month history of anorexia, weight loss, generalized abdominal pain, dysphagia, and watery diarrhea. There were no associated comorbidities. On physical examination, there was pallor, abdominal examination revealed a vague mass which was firm and tender.

Baseline full blood count revealed hemoglobin 13.0 g/dl (13.5-17.5), white cell count 7.48 x10^9^/L (4-11), neutrophils 60% (40-75), lymphocytes 22.0% (20-45%), monocytes 14.2% (2-10%), eosinophils 3.2 % (1-6), basophils 0.1% (0.1-1%), platelet count 809 x10^9^/L (150-400). Other results included Erythrocyte Sedimentation Rate (ESR) 35mm (<25), creatinine 0.90mg/dl (0.7-1.2), LDH 436 IU (<225).

**Fig.1:**
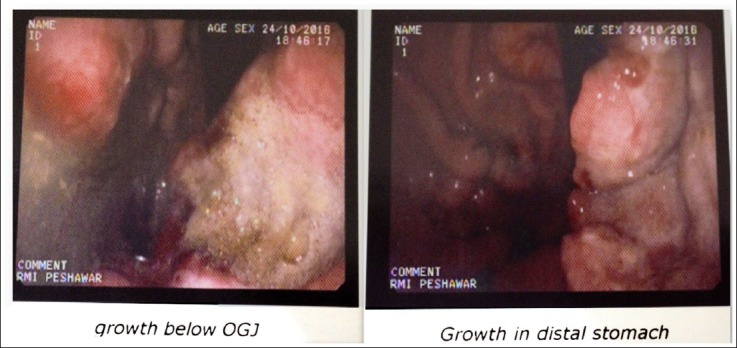
Showing Endoscopic finding of the patient.

The upper gastrointestinal endoscopy revealed a large area of mucosal growth with central ulceration. Another area of mucosal growth over lesser curvature in distal stomach. (Fig.1) Carbohydrate antigen (CA) 19-9 and carcinoembryonic antigen (CEA) were within normal limits. Tests for HIV were negative on two occasions.

Two specimens were taken for biopsy. Specimens showed High-grade Non-Hodgkin B-cell Lymphoma, Morphological and immunohistochemistry feature favors Burkitt’s lymphoma. (Fig. 2A and 2B) Immunohistochemistry shows, CD20: strong and diffusely positive for the tumor, CD3: negative for tumor cells, Mib-1: 98%-99%. CD10: Positive for tumor cells. BCL2L: Negative for tumor cells. (Fig. 3A and 3B)

**Fig.2A:**
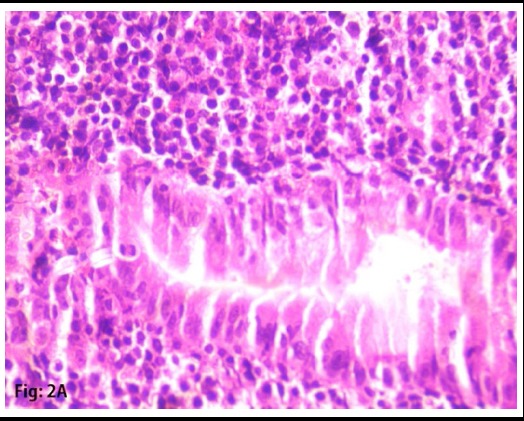
H and E section at 40x magnification showing atypical lymphoid cells surrounding and infiltrating gastric glands.

**Fig.2B:**
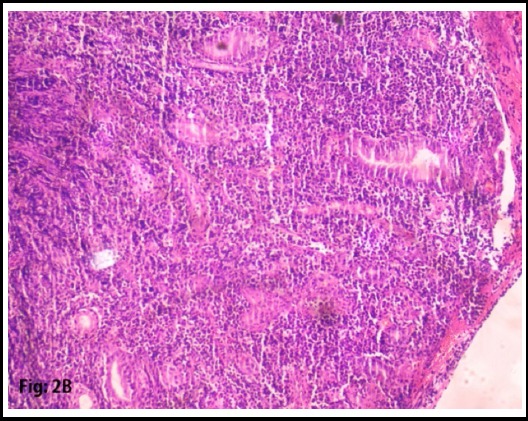
H and E section at 10 x magnification showing diffuse sheet of atypical lymphoid. Cells involving gastric mucosa.

**Fig.3A:**
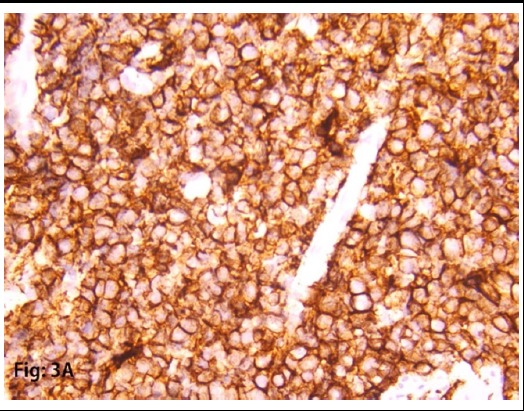
Strong and diffuse positivity of CD20 in tumor cells.

**Fig.3B:**
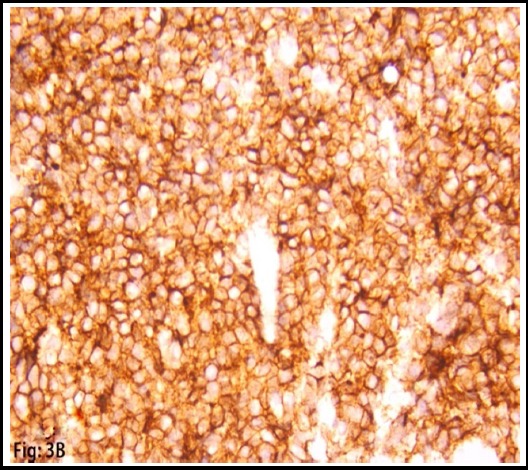
Expression of CD10 in tumor cells.

Patient was treated using a third-generation chemotherapy protocol for NHL using Cyclophosphamide, Ifosfamide, Doxorubicin, Etoposide, Ara-C, Methotrexate, and Vincristine with prophylactic intrathecal Methotrexate. Complete remission after 4 cycles was documented on repeat UGIE, CT scan.

## DISCUSSION

Burkitt’s lymphoma being one of the most aggressive forms of B-cell NHL, with replication approaching 100% has three clinical forms; endemic, sporadic and immunodeficiency associated. The endemic variant is common in Africa, the sporadic variant is present in the U.S and Western Europe, and immunocompromised variant occurs mainly in HIV patients. The sporadic variant comprises 30% of pediatric lymphomas and less than 1% of adult NHL.^6^

The most frequently affected site outside of lymph node involvement is the gastrointestinal tract (30-50%).^7^ Primary gastrointestinal lymphoma is rare. Secondary involvement of the gastrointestinal tract is common in lymphoma. Primary gastrointestinal lymphoma presents with symptoms localized to the GI tract or predominating mainly in the GI tract.

The primary involvement of BL or a small non-cleaved cell lymphoma in the GI tract, although rare, has been reported in the literature. Despite gastric lymphomas being more common than intestinal lymphomas, the primary gastric involvement is extremely rare in BL. For non-endemic Burkitt’s lymphoma, the gastrointestinal tract is the most common site, followed by retro peritoneal, kidney, ovary, and testes respectively.^8^ Burkitt’s lymphoma of stomach is an exceptionally rare disease in adults.

The exact mechanism leading to formation of Burkitt’s lymphoma is not yet known. The Epstein-Barr virus has been implicated to have involvement as it can be found in 25-40% immunodeficiency variant cases of Burkit’s lymphoma. Normal gene expression and translation process of cellular microRNA has been shown to be interfered with by Epstein-Barr virus interaction with the cellular microRNA.^9^ Burkitt’s lymphoma affects patients with CD4 T cell counts greater than 200/mm^3^, which may suggest that immunity does not have a role in the matter, however, in our case study it was not the case.

Burkitt’s lymphoma is a very aggressive malignancy and one of the fastest growing amongst human malignancies.^6^ It requires immediate and aggressive intervention. Fortunately, it does respond to aggressive chemotherapy^10^ regardless of it being a very rapidly growing malignancy, chemotherapy being the gold standard treatment for it. Tumor lysis syndrome which is a complication of rapid, massive and acute destruction of the tumor cells can occur during initial chemotherapy and one should remain wary of that fact. The more extended the disease, the more the chances it will get complicated and thus harder to treat.

## CONCLUSION

Gastric Burkitt’s lymphoma is an aggressive tumor with high proliferation; we are yet to find the optimal treatment to this disease to help its poor prognosis. We have been able to hold it at bay by aggressive chemotherapy, mainly derived from pediatric treatment regiments, unspecific to Burkitt’s. Chemotherapy, rituximab and prophylactic CNS treatment should be included as part of the treatment regimens. Early diagnosis with aggressive and early treatment can give very good long term survival rates for the patients, reaching around 70-80%. However, letting the disease advance and get into older age groups can be very rapidly lethal for the patients. It is a disease which deserve great attention and as we are at the edge of breaking through into its treatment. An effort made into early detection can make a vast difference in outcome for the patient.
